# Time usage analysis according to occupational area and satisfaction level in family caregivers of dementia patients

**DOI:** 10.7717/peerj.15178

**Published:** 2023-04-14

**Authors:** Woo-Hyuk Jang, Jong-Sik Jang, Jong-Hwi Park

**Affiliations:** Department of Occupational Therapy, Kangwon National University, Samcheok-si, Gangwon-do, South Korea

**Keywords:** Dementia family, Occupational area, Time usage, Life satisfaction, Leisure satisfaction

## Abstract

**Background:**

This study was designed to investigate the difference between the family caregivers of dementia patients (hereafter referred to as dementia family) and the non-family caregivers of dementia patients (hereafter referred to as non-dementia family) in terms of time usage.

**Methodology:**

A total of 102 dementia families who responded to the ‘time use survey’ in 2019 were enrolled in the study. 101 non-dementia families include families who did not respond to the ‘dementia’ item, and simple random sampling was performed. Time usage according to occupational area and satisfaction level were analyzed based on the Occupational Therapy Practice Framework-Fourth Edition (OTPF-4). Statistical analyses were completed using IBM SPSS 25. The data was analyzed by using frequency analysis and independent two-sample *t*-test. A level of *p* < 0.05 was used as a cut-off for statistical significance.

**Results:**

As for the time consumption by occupational area of dementia families and non-dementia families, dementia families spent more time than non-dementia families in instrumental daily life activities. The increase in the time for instrumental activities of daily living, including the time for caring for dementia patients, may lead to changes in time use for members of the family with dementia. By comparing the time usage by occupational area according to gender within the dementia families, it was possible to find out the difference between male and female instrumental daily activities and health care time use. The difference in time use according to gender showed that women took on more caring roles than men, and actually spent more time than men.

**Conclusion:**

The amount of time used between the dementia family and the non-dementia family differed according to the group and gender. These results suggest that dementia can cause changes in the time usage of dementia family. Therefore, this study recognizes the need for efficient use of time for dementia families and suggests that there is a need for a balanced use of time according to gender.

## Introduction

The percentage of elderly population aged 65 years or older in Korea is 15.7% as of 2020, and it is expected to be 20.3% by 2025, resulting in a super-aged society (Statistics Korea, 2020a). This population aging is expected to cause an acute increase in the elderly dementia patient population (Ministry of Health & Welfare, 2020). The prevalence rate of dementia in the elderly population aged 65 years or older was 0.75 million (10.16%) in 2018. It is expected to be 1.36 million (10.48%) by 2030 and 3.02 million (15.91%) by 2050, thereby showing a twofold increase every 20 years (Statistics Korea, 2019; Statistics Korea, 2020b).

Impairment of the neurons in the brain attributes to the deficiency in various fields of cognition, behavioural changes, and personality changes in dementia (Sung et al., 2013). In addition, the impairment of cognitive function leads to multiple issues concerning daily life and social life (Kim, 2019). Therefore, dementia patients require constant care and attention from others (Sung et al., 2013), presenting more physical and mental burden on the family caregivers of dementia patients (hereafter referred to as dementia family) compared to other diseases (Suh & An, 2009). This is emphasized by a survey, which showed that dementia patients require caretaking for an average of 5 h per day per person (Kim et al., 2016). In order to take constant care of the elderly dementia patients, dementia families need to adjust their daily life according to the patients’ schedule, which disrupts their time allocation and performance of daily living (Kim, Kwon & Lee, 2018). The limitation of time usage in dementia families leads to the reduction or severance of their social relationships, which has a negative impact on the caregiving process (Ryu et al., 2018). As the amount of time dementia families spends caring for the dementia patient increases, the quality of life is negatively affected (Hazzan et al., 2022).

Time is a component of activities of daily living (ADLs) (Jeon, 2011), and an adequate time usage signifies that the activities of daily living are being smoothly performed (Bejerholm, 2010). Adequate time allocation leads to a balanced daily life, thus increasing life satisfaction levels (American Occupational Therapy Association, 2020). However, due to the limitations of the time component, a person with multiple roles is confronted with difficulties in time usage (Hong & Kim, 2014). Therefore, it is important for an individual to choose a meaningful occupation as a performance with objectives and significance (American Occupational Therapy Association, 2020). Furthermore, time is utilized as a tool for the study of occupations due to the close relationship (Hunt & McKay, 2015). Hence, Statistics Korea has conducted the ‘time use survey’ every 5 years since 1999, researching the 24-hour occupations in various populations.

Multiple studies have been conducted based on this ‘time use survey’, analyzing the time usage according to the occupational area (Hong & Lee, 2010; Hong & Kim, 2014; Bak & Cha, 2020; Bak & Kim, 2020; Kim, Hong & Park, 2017). First of all, a study that analyzed the data from the ‘time use survey’ in 2004 classified the daily activities into occupational areas and compared their time usage (Hong & Lee, 2010). The results showed that the time usage for the instrumental activities of daily living (IADLs), including the caregiving area for the family and the community, was the highest in early adolescence, while the time usage for rest and sleep was the highest in senescence. In addition, the male subjects showed high time usage for work in adulthood, while the female subjects showed high time usage for IADLs in adulthood. A study that analyzed time usage in mothers of infants who responded to the ‘time use survey’ in 2009 showed that sleep was the largest occupational area, followed by work, leisure, infant care, and activities of daily living (ADLs) (Hong & Kim, 2014). A study that analyzed the time usage according to occupational area in disabled adolescents based on the ‘time use survey’ showed that the time usage for rest and sleep was the highest, followed by ADLs, education, leisure, social participation, and entertainment (Bak & Cha, 2020). A study that analyzed time usage in disabled elderly based on the ‘time use survey’ in 2014 classified the occupational areas into sleep activities, daily activities, work activities, and leisure activities, and the disabled elderly showed the highest time usage for sleep activities, followed by leisure activities (Bak & Kim, 2020). A study that researched the changes in time usage according to the occupational area by age of the population included in the ‘time use survey’ from 2004, 2009, and 2014 showed that, as ‘time use survey’ progressed, the overall time usage for leisure increased in ages 60 or older, and the time usage for work during weekdays and time usage for leisure during weekends increased in ages 40 to 59 (Kim, Hong & Park, 2017). Elderly people who experienced cerebrovascular accident showed less time usage for sleep and IADLs compared to those who did not experience a cerebrovascular accident (McKenna et al., 2009). In addition, people who did not experience a cerebrovascular accident undertook more roles compared to those who did, and life satisfaction level was found to increase in proportion to the number of roles that an individual was performing (McKenna et al., 2009). Even though there have been numerous studies conducted on the subject of time usage, these previously mentioned pilot studies classify occupational areas according to the Occupational Therapy Practice Framework-Third Edition, which was published before 2020 (Hong & Lee, 2010; Hong & Kim, 2014; Bak & Cha, 2020; Bak & Kim, 2020; Kim, Hong & Park, 2017; McKenna et al., 2009). Furthermore, no studies have been conducted so far that classify occupational areas according to the Occupational Therapy Practice Framework-Fourth Edition (OTPF-4), which was newly published in 2020.

Pilot studies were also conducted in order to determine time usage in dementia families (Taylor, Kuchibhatla & Ostbye, 2008; Hajek et al., 2015; Kim & Kim, 2009). A study reported reduced caregiving time of the patients’ spouses in the cases of dementia patients who take physical care of themselves and who have less limitations of ADLs (Taylor, Kuchibhatla & Ostbye, 2008). Caregiving time provided by the dementia family or acquaintances increased in proportion to the severity of dementia and the age of the patients (Hajek et al., 2015). A study that compared the caregiving time of dementia families showed that life satisfaction level was higher when the caregiving time was 4∼9 h compared to more than 10 h (Kim & Kim, 2009). Studies conducted on dementia families were centered around caregiving time, and not focused on time usage. Moreover, no distinction was made between the time usage of weekdays and weekends in dementia families and non-family caregivers of dementia patients (hereafter referred to as non-dementia family). There were no studies that analyzed time usage of dementia family based on the occupational areas according to the latest OTPF-4 (Taylor, Kuchibhatla & Ostbye, 2008; Hajek et al., 2015; Kim & Kim, 2009).

In the previous study, various groups were investigated for time use according to the work area, but no study was conducted on dementia families, nor did a study to find out the difference in time use by comparing with dementia families and non-dementia families. Therefore, this study was designed to classify occupational areas according to the OTPF-4 edition in determining the time usage of activities from the ‘time use survey’ data of 2019. Additionally, in order to visualize the everyday life of the study subjects more closely, the time usage according to occupational area, life satisfaction, and leisure satisfaction levels were studied based on weekdays in dementia families and non-dementia families.

## Materials & Methods

### Study subjects

**Figure 1:**
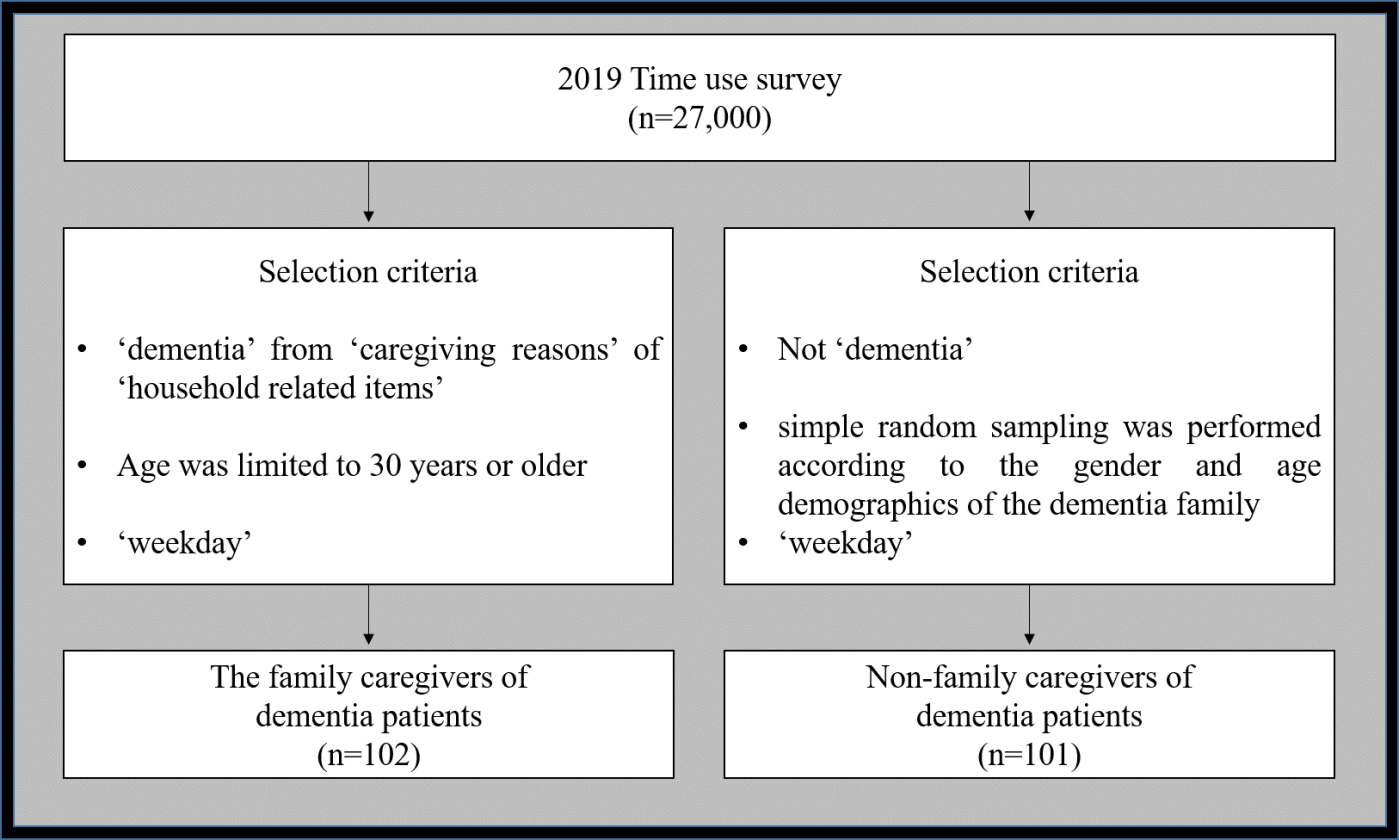
Criteria for selection of study subjects.

This study utilized the data from the ‘time use survey’ conducted by Statistics Korea in 2019 and performed secondary data analysis (Sung, Kim & Lee, 2012). From 27,000 subjects who responded to the survey, the family caregivers of dementia patients (hereafter referred to as dementia family), who responded to ‘dementia’ from ‘caregiving reasons’ of ‘household related items’, do not only include the main caregiver, but also the entire household that takes care of the elderly dementia patient. Subject age was limited to 30 years or older, excluding ages 10∼29 which contains a large group of students. Non-family caregivers of dementia patients (hereafter referred to as non-dementia family) include families who did not respond to the ‘dementia’ item, and simple random sampling was performed according to the gender and age demographics of the dementia family. Additionally, the subjects who responded to ‘Saturday’ and ‘Sunday’ items were excluded, and only the subjects who responded to ‘weekday’ items were included (Fig. 1). This study was approved by the Kangwon National University Institutional Review Board (KWNUIRB-2021-07-001) prior to commencement.

### Study tools

#### 2019 time use survey

‘Time use survey’ is conducted in order to investigate the average daily life of Korean people by collecting data on the current time usage of 24 h and time awareness, performed nationwide every 5 years since 1999 (Sung, Kim & Lee, 2012). The subjects recorded their activities in a time log every 10 min for 2 days (48 h). If an activity lasted for more than 5 min, it is considered to have lasted for 10 min, and if multiple activities were performed within 10 min, the activity that lasted for the longest period of time was recorded. These activities were classified into 9 large categories, 45 medium categories, and 153 small categories. Time was converted into minutes based on a single day (24-hour period), analyzed, and provided.

#### Satisfaction level

Life satisfaction and leisure satisfaction levels were determined by the items ‘How do you feel about your everyday life in general?’ and ‘How do you feel about your leisure time in general?’ from the ‘time use survey’ conducted by Statistics Korea in 2019. A 5-point Likert scale was used to evaluate the life satisfaction and leisure satisfaction levels based on ‘very satisfactory’ (1 point) to ‘very unsatisfactory’ (5 points). The scores were reverse coded so that the high scores were interpreted as high satisfaction levels.

### Study process

#### Classification of activities based on occupational areas

**Figure 2:**
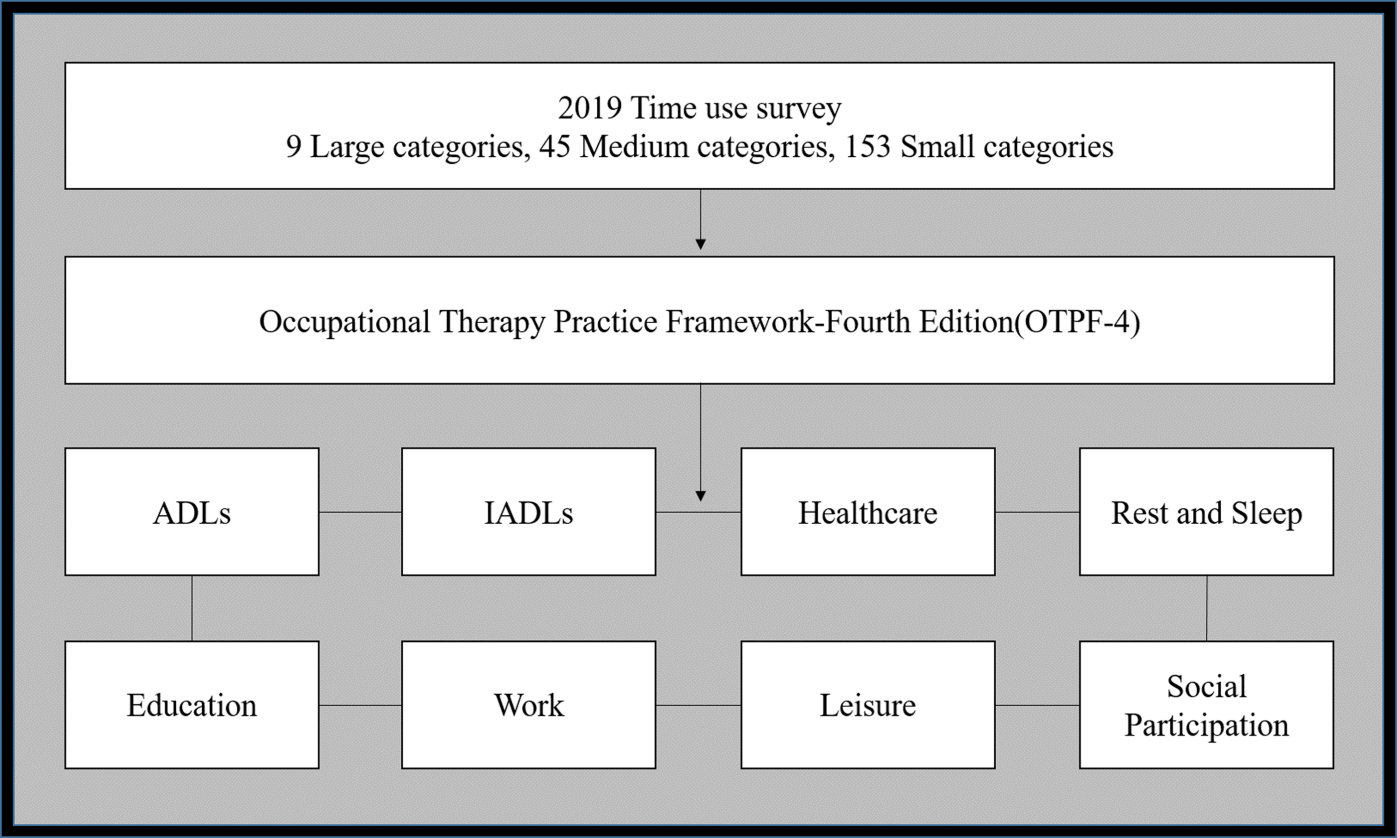
Classification of activities based on occupational areas. The classification criteria of the time sue survey were reclassified as those of the Occupational Therapy Practice Framework-Fourth Edition (OTPF-4). The area used is activities of daily living (ADLs), instrumental activities of daily living (IADLs), healthcare, rest and sleep, education, work, leisure, and social participation.

The ‘time use survey’ of 2019 classified the activities of the subjects into 9 large categories, 45 medium categories, and 153 small categories. However, this study re-classified and analyzed the activities into areas of activities of daily living, instrumental activities of daily living, healthcare, rest and sleep, education, work, leisure, and social participation based on the occupational areas provided in the Occupational Therapy Practice Framework-Fourth Edition (OTPF-4) (American Occupational Therapy Association, 2020) (Fig. 2).

### Statistical analysis

The IBM SPSS 25.0 program (IBM, Chicago, IL, USA) was used to analyze the data in this study, and the significance level was 0.05. Frequency analysis was used on the general characteristics of the subjects, and independent *t*-test was performed in order to analyze the difference between time usage in various groups, life satisfaction and leisure satisfaction levels, and gender difference of dementia families. A level of *p* < 0.05 was used as a cut-off for statistical significance.

## Results

### General characteristics of study subjects

The general characteristics of the dementia family are as follows. In regard to gender, 55 (53.9%) subjects were females and 47 (46.1%) subjects were males. In regard to age, 56 (54.9%) subjects were 60 years or older, and 30 (29.4%) subjects were between 50∼59 years old, accounting for most of the age group. In regard to marital status, 77 (75.5%) subjects were married and 13 (12.7%) subjects were unmarried. In regard to education level, 39 (38.2%) subjects have received a high school diploma, which was the most common educational level. In regard to job status, 57 (55.9%) subjects were out of work and 45 (44.1%) subjects were working (Table 1).

The general characteristics of the non-dementia family are as follows. In regard to gender, 51 (50.5%) subjects were males and 50 (49.5%) subjects were females. In regard to age, 65 (64.4%) subjects were 60 years or older and 23 (22.8%) subjects were between 50∼59 years old, accounting for most of the age group. In regard to marital status, 74 (73.3%) subjects were married and 18 (17.8%) subjects were in bereavement. In regard to education level, 34 (33.7%) subjects have received a high school diploma, which was the most common educational level. In regard to job status, 62 (51.2%) subjects were working and 39 (48.8%) subjects were out of work (Table 1).

### Differences in time usage according to occupational area

Differences in time usage according to occupational area was compared on a 24-hour basis, and the results showed that dementia families (283.24 min) used a longer period of time than non-dementia families (223.27 min) in the area of IADLs ( *t* = 2.501, *p* = 0.013). Other areas did not show a statistically significant difference (Table 2).

**Table 1:** General characteristics of dementia family and non-dementia family. Characteristics are gender, age, marital status, education level, job status.

Characteristics		Dementia family (*n* = 102)		Non-dementia family (*n* = 101)	
Gender	Male N (%)	47 (46.1)		51 (50.5)	
	Female N (%)	55 (53.9)		50 (49.5)	
		M/F (%: %)	*n* (%)	M/F (%: %)	*n* (%)
Age	30∼39	4: 2 (67: 33)	6 (5.9)	3: 1 (75: 25)	4 (4.0)
	40∼49	2: 8 (20: 80)	10 (9.8)	2: 7 (22: 78)	9 (8.9)
	50∼59	21: 9 (70: 30)	30 (29.4)	18: 5 (78: 22)	23 (22.8)
	60∼	20: 36 (36: 64)	56 (54.9)	28: 37 (43: 57)	65 (64.4)
Marital status	Single	7: 6 (54: 46)	13 (12.7)	3: 0 (100: 0)	3 (3.0)
	Married	33: 44 (43: 57)	77 (75.5)	45: 29 (61: 39)	74 (73.3)
	Divorced	7: 0 (100: 0)	7 (6.9)	2: 4 (33: 67)	6 (5.9)
	Bereavement	0: 5 (0: 100)	5 (4.9)	1: 17 (6: 94)	18 (17.8)
Education level	Elementary school or lower	4: 25 (14: 86)	29 (28.4)	2: 25 (7: 93)	27 (26.8)
	Middle school	3: 10 (23: 77)	13 (15.7)	9: 4 (69: 31)	13 (12.9)
	High school	25: 14 (64: 36)	39 (38.2)	20: 14 (59: 41)	34 (33.7)
	College	4: 4 (50: 50)	8 (7.8)	11: 3 (79: 21)	14 (13.9)
	University	5: 8 (38: 62)	13 (12.7)	9: 4 (69: 31)	13 (12.9)
	Master’s degree	4: 0 (100: 0)	4 (3.9)	–	–
	Doctor’s degree	2: 0 (100: 0)	2 (2.0)	–	–
Job status	Yes	20: 25 (44: 66)	45 (44.1)	40: 22 (65: 35)	62 (51.2)
	No	27: 30 (47: 53)	57 (55.9)	11: 28 (28: 72)	39 (48.8)

**Notes.**

M Male F Female

### Differences in time usage of IADLs

Differences in time usage between the subcategories of IADLs from occupational area time usage were compared, and the results showed that dementia families (46.26 min) used more time than non-dementia families (1.39 min) in the area of ‘caring for adult families and household members with long-term care needs’ (*t* = 4.467, *p* < 0.001). In areas other than ‘caring for adult families and household members with long-term care needs’, no statistically significant differences were observed (Table 3).

**Table 2:** Difference in time usage according to occupational area. Characteristics are ADLs, IADLS, health management, rest and sleep, education, work, leisure, social participation. There is a difference in time usage between the two groups in IADLs (Unit: Minute).

Characteristics	Dementia family (*n* = 102)		Non-dementia family (*n* = 101)		*t*	*p*-value
	M ± SD	%	M ± SD	%		
ADLs	193.43 ± 62.18	13.43	198.42 ± 62.99	13.78	−0.567	0.571
IADLs	283.24 ± 19079	19.67	223.27 ± 147.92	15.50	2.501	0.013^*^
Health management	56.37 ± 98.35	3.91	56.63 ± 71.32	3.93	−0.022	0.983
Rest and sleep	496.86 ± 111.44	34.50	495.55 ± 98.61	34.41	0.089	0.929
Education	7.55 ± 44.20	0.52	5.35 ± 41.73	0.37	0.365	0.715
Work	172.26 ± 215.93	11.96	207.23 ± 228.51	14.39	−1.122	0.263
Leisure	175.88 ± 158.52	12.21	195.94 ± 139.92	13.61	−0.955	0.341
Social participation	54.41 ± 75.33	3.78	57.62 ± 81.01	4.00	−0.293	0.770

**Notes.**

* *p* < 0.05

**Table 3:** Differences in time usage of IADLs. There is a difference in time usage between the two groups in ‘Caring for adult families and household members with long-term care needs’ (Unit: Minute).

Characteristics	Dementia family (*n* = 102)	Non-dementia family (*n* = 101)	*t*	*p*-value
	M ± SD	M ± SD		
Food preparation	69.51 ± 83.07	56.24 ± 63.57	1.277	0.203
Clothing management	14.80 ± 28.76	9.90 ± 22.43	1.354	0.177
Cleaning and tidying up	22.65 ± 28.73	21.68 ± 35.67	0.212	0.832
Housing and household goods management	1.18 ± 7.22	10.69 ± 63.63	−1.501	0.135
Vehicle management	0.00	0.99 ± 8.19	−1.222	0.223
Caring for pets and plants	1.37 ± 7.45	0.89 ± 4.27	0.564	0.573
Purchasing goods and services	12.35 ± 27.80	9.80 ± 19.80	0.752	0.453
Home management	2.25 ± 13.19	3.07 ± 15.80	−0.399	0.690
Caring for children under the age of 10	0.00	5.35 ± 32.70	−1.651	0.100
Caring for minors between the ages of 10 and 18	2.45 ± 11.38	1.68 ± 9.60	0.519	0.604
Caring for adult families and household members with long-term care needs	47.65 ± 103.16	1.39 ± 13.93	4.467	0.000^***^
Caring for independent adult families and members of the household	1.27 ± 7.79	1.19 ± 7.11	0.082	0.934
Religious activity	9.90 ± 37.51	3.76 ± 15.61	1.520	0.130
Ceremonial activity	0.10 ± 0.99	0.00	0.995	0.321
Locomotion	97.75 ± 87.47	96.63 ± 86.40	0.091	0.928

**Notes.**

*** *p* < 0.001

### Differences in time usage according to occupational area by gender of the dementia family

Differences in time usage according to occupational area by gender of the dementia family were analyzed, and the results showed that in the area of IADLs, the female subjects (326.18 min) used a longer period of time than the male subjects (232.98 min) by a difference of 93.2 min (*t* =  − 2.524, *p* = 0.013). In the area of health management, the male subjects (87.454 min) used a longer period of time than the female subjects (29.82 min) by a difference of 57.63 min (*t* = 3.070, *p* = 0.003). In areas other than IADLs and health management, no statistically significant differences were observed (Table 4).

**Table 4:** Differences in time usage in occupational area according to the gender of the dementia family. There is a difference according to gender of the dementia family in the IADLs area and the health management area (Unit: Minute).

Characteristics	Male (*n* = 47)	Female (*n* = 55)	*t*	*p*-value
	M ± SD	M ± SD		
ADLs	192.34 ± 51.97	194.36 ± 70.21	−0.163	0.871
IADLs	232.98 ± 179.93	326.18 ± 190.93	−2.524	0.013^*^
Health management	87.45 ± 128.12	29.82 ± 50.50	3.070	0.003^**^
Rest and sleep	474.26 ± 516.18	516.18 ± 113.11	−1.919	0.058
Education	1.49 ± 10.21	12.73 ± 59.21	−1.284	0.202
Work	203.62 ± 239.37	145.46 ± 191.86	1.362	0.176
Leisure	181.70 ± 162.42	170.91 ± 156.45	0.341	0.734
Social participation	66.17 ± 99.75	44.36 ± 43.75	1.466	0.146

**Notes.**

* *p* < 0.05

** *p* < 0.01

### Differences in time usage for IADLs according to the gender of the dementia family

Differences in time usage for subcategories of IADLs according to the gender of the dementia family were analyzed, and the results showed that the female subjects (70.36 min) used a longer period of time than the male subjects (21.06 min) in the area of ‘caring for adult families and household members with long-term care needs’ by a difference of 49.3 min (*t* =  − 2.466, *p* = 0.015). The female subjects (86.73 min) used a longer period of time than the male subjects (49.36 min) in the area of ‘food preparation’ by a difference of 37.37 min (*t* =  − 2.313, *p* = 0.023). Likewise, the female subjects (23.82 min) used a longer period of time than the male subjects (19.56 min) in the area of ‘clothing management’ (*t* =  − 3.624, *p* < 0.001) (Table 5).

**Table 5:** Differences in IADLs lifetime use according to the gender of the dementia family. There is a difference in time usage in three areas according to gender of the dementia family (Unit: Minute).

Characteristics	Male (*n* = 47)	Female (*n* = 55)	*t*	*p*-value
	M ± SD	M ± SD		
Food preparation	49.36 ± 83.94	86.73 ± 79.05	−2.313	0.023^*^
Clothing management	4.26 ± 13.63	23.82 ± 34.77	−3.624	0.000^***^
Cleaning and tidying up	17.02 ± 29.19	27.46 ± 27.70	−1.850	0.067
Housing and household goods management	1.92 ± 9.70	0.55 ± 4.05	0.955	0.342
Vehicle management	0.00	0.00		
Caring for pets and plants	0.85 ± 5.84	1.82 ± 8.63	−0.652	0.516
Purchasing goods and services	11.28 ± 29.01	13.27 ± 26.95	−0.360	0.720
Home management	0.21 ± 1.46	4.00 ± 17.81	−1.453	0.149
Caring for children under the age of 10	0.00	0.00		
Caring for minors between the ages of 10 and 18	1.28 ± 6.12	3.46 ± 14.43	−0.963	0.338
Caring for adult families and household members with long-term care needs	21.06 ± 57.83	70.36 ± 126.15	−2.466	0.015^*^
Caring for independent adult families and members of the household	2.55 ± 11.32	0.18 ± 1.35	1.542	0.126
Religious activity	13.83 ± 50.37	6.55 ± 21.10	0.977	0.331
Ceremonial activity	0.00	0.18 ± 1.35	−0.924	0.358
Locomotion	109.36 ± 105.26	87.82 ± 68.22	1.243	0.217

**Notes.**

* *p* < 0.05

*** *p* < 0.001

### Differences in health management time usage according to the gender of the dementia family

Differences in the subcategories of health management time usage according to the gender of the dementia family were analyzed, and no significant results were observed in the areas of ‘personal health care’ and ‘sports and leisure sports’ (Table 6).

**Table 6:** Differences in health management time usage according to the gender of the dementia family. There is no difference between the two groups according to gender, but there is a slight difference in the sports area.

Characteristics	Male (*n* = 47)	Female (*n* = 55)	*t*	*p*-value
	M ± SD	M ± SD		
Personal health care	41.06 ± 116.83	16.00 ± 40.63	1.490	0.139
Sports and leisure sports	21.49 ± 54.81	6.18 ± 15.21	1.985	0.050

### Differences in life satisfaction and leisure satisfaction levels

Differences in life satisfaction and leisure satisfaction levels between dementia families and non-dementia families were analyzed, and both groups showed a similar score, thereby resulting in no statistically significant difference between the groups (Table 7).

**Table 7:** Difference between life satisfaction and leisure satisfaction. There is a difference between the two groups in life satisfaction.

Characteristics	Dementia family (*n* = 102)	Non-dementia family (*n* = 101)	*t*	*p*-value
	M ± SD	M ± SD		
Life satisfaction	2.94 ± 1.18	2.94 ± 0.90	0.004	0.997
Leisure satisfaction	2.98 ± 1.14	3.00 ± 0.98	−0.131	0.896

Differences in life satisfaction and leisure satisfaction levels according to the gender of the dementia family were analyzed, and the female and male subjects showed a similar score, thereby resulting in no statistically significant difference between the groups (Table 8).

**Table 8:** Difference between life satisfaction and leisure satisfaction according to the gender of the dementia family. There was no difference in time usage in satisfaction according to gender of the dementia family. Although there is no difference, the overall score is below 3 points.

Characteristics	Male (*n* = 47)	Female (*n* = 55)	*t*	*p*-value
	M ± SD	M ± SD		
Life satisfaction	2.87 ± 1.055	3.00 ± 1.28	−0.545	0.587
Leisure satisfaction	3.13 ± 1.08	2.86 ± 1.19	1.206	0.231

## Discussion

This study was based on the ‘time use survey’ of 2019, and the data from weekdays was selected to represent everyday life more closely. Occupational area was classified according to the Occupational Therapy Practice Framework-Fourth Edition (OTPF-4). Time usage according to occupational area, life satisfaction level, and leisure satisfaction level were investigated in family caregivers of dementia patients (dementia family) during weekdays, and the difference between dementia family and non-family caregivers of dementia patients (non-dementia family) was investigated in time usage according to occupational area, life satisfaction level, and leisure satisfaction level during weekdays.

As for the general characteristics of the subjects, most of the subjects were 60 years or older in both groups, followed by ages 50∼59. This is considered to be due to the recent trend of familial caregiving performed by the spouse rather than the child or daughter-in-law (Sung, Kim & Lee, 2012). However, in the case of dementia families, there were more female subjects (53.9%) than male subjects (46.1%), compared to non-dementia families, which did not show a definite gender difference between the male subjects (50.5%) and female subjects (49.5%). This shows that caregiving by female spouses increased as the number of male dementia patients increased (Kwon & Hong, 2019). As for marital status, both groups showed that the highest number of subjects were married. However, only the non-dementia family showed the second highest number of subjects who were in bereavement, which suggests that the chances of caregiving for dementia patients was low due to the absence of the spouse. Both groups showed educational level in the order of ‘high school diploma’ and ‘elementary school or lower’, and while there is a difference in the rest of the ranking, it is insufficient to be considered as a source for the objective analysis of educational level difference between the groups. Job status showed that while only 44.1% of dementia families were working, 51.2% of non-dementia families were working. This indirectly suggests the financial burden that the dementia families are confronted with, which is supported by a pilot study that investigated the financial burden of dementia families (Kil & Cho, 2020).

Dementia families used more time than non-dementia families in the area of instrumental activities of daily living (IADLs). This result shows that dementia families use more time for IADLs, which are activities that provide care for the family and the society, compared to non-dementia families. In contrast to a pilot study, in which all subjects who responded to the ‘time use survey’ were enrolled and the results showed a difference by gender and age in time usage according to occupational area (Hong & Lee, 2010; Kim, Hong & Park, 2017), this study showed a significant difference in the area of IADLs even though there was no obvious difference of the gender and age between the groups. In detail, dementia families used more time in the area of ‘caring for adult families and household members with long-term care needs’. This is considered to be due to the longer caregiving time of dementia families for the elderly dementia patients (Kim, Kwon & Lee, 2018). This excessive use of time for caring activities creates a time imbalance, which can worsen quality of life and cause health problems (Jeon, 2011). Because Dementia families have to take care of people with dementia, it becomes difficult to meet neighbors or friends, they can no longer engage in hobbies, and they are unable to attend family events and are often disconnected from society (Ryu et al., 2018). In order to solve this problem, support at the national level is needed, and adaptation to care is needed through the provision of positive meaning of care within the dementia families (Kil & Cho, 2020).

Investigation of the gender difference in dementia families showed that the female subjects used more time than the male subjects in the area of IADLs, and the male subjects used more time than the female subjects in the area of health management. This is similar to a study which showed that the female subjects used more time than the male subjects in the area of IADLs in the age group of 27 years or older (Hong & Lee, 2010), and the difference in the area of health management is considered to be due to more active participation in sports of the male subjects compared to the female subjects (Heritage, 2013). The subcategories of IADLs for which the female subjects used more time than the male subjects were ‘caring for adult families and household members with long-term care needs’, ‘food preparation’, and ‘clothing management’, and there were no significant differences in the subcategories of health management for which the male subjects used more time than the female subjects. This is similar to the result of a pilot study that showed female subjects in general used more time than the male subjects in the area of household management in the age group of 19 years or older (Hong & Lee, 2010). Studies showing that women spend more time on housework than men and that their happiness level is lower (Giurge, Whillans & Yemiscigil, 2021), the imbalance of time devoted to household chores leads to an equal distribution of household chores when the husband’s days off lead to an equal distribution of household chores (Álvarez & Miles-Touya, 2019). However, there is a difference that the females of the dementia family use more time in caregiving for family members, as well as household management. In addition, the females of the dementia family take the role of main caregiver and provide direct caregiving due to the traditional Confucianism, but the males did not undergo role changes due to caregiving (Yih, Kim & Yi, 2004).

There was no significant difference in satisfaction scores between dementia families and non-dementia families. This is in contrast to a pilot study, which indicated that dementia families feel fatigue resulting from the caregiving of dementia patients, and they also feel the change in their lives (Ryu et al., 2018). This is supported by the results of a study which showed that the time used by dementia families in caregiving of dementia patients was utilized by the non-dementia families for activities that affect life satisfaction levels (Choi, 2018). Furthermore, there was no significant gender difference in the life satisfaction and leisure satisfaction levels of dementia families. This is in accordance with a pilot study that there is no gender difference in life satisfaction and support burden (Lee, Cho & Kim, 2009; Lee, Lee & Lee, 2015). However, the below-average satisfaction levels in both genders imply that further ameliorations are necessary.

The limitations of this study included the insufficient investigation of the younger generation because the subjects in their teens and twenties were excluded based on their main time usage in schools, and that the time usage of weekends were excluded. Additionally, factors such as the total caregiving period of the dementia family for the dementia patient, the severity of dementia symptoms, and period of time following the onset of the dementia were not considered. However, it is of importance that this study investigated the difference between the time usage of dementia families and non-dementia families according to occupational area by analyzing not only the time usage of the main caregiver, but also the entire household members, as well as the difference in time usage between the gender groups of dementia families. Furthermore, this study implies that dementia can change the time usage of dementia families, and that the members of the dementia family, including the main caregiver, should be aware of the imbalance in time usage. In addition, various types of external support are deemed necessary in order to overcome these issues.

## Conclusions

The amount of time used by occupational area between the dementia families and the non-dementia families differed according to group and gender. This difference has the potential to be a factor that can cause time imbalance in the caregiver’s time use. In addition, it was shown that dementia causes changes in the time use of caregivers, suggesting the need for interventions in the time use of caregivers due to dementia.

These results are anticipated to aid in the awareness for the necessity of efficient time usage in dementia families, and also to be utilized as a basis for the measures to achieve balanced time usage between the gender groups. In addition, in future research, it is necessary to study the use of time not only on weekdays but also on holidays, the difference in time use according to the difference in roles within the dementia family, the change in roles and the effect on life according to the difference in time use.

##  Supplemental Information

10.7717/peerj.15178/supp-1 Data S1 Raw data: 2019 time use survey The time usage survey is conducted in order to investigate the average daily life of Korean people by collecting data on the current time usage of 24 hours and time awareness, performed nationwide every 5 years since 1999. The subjects recorded their activities in a time log every 10 minutes for 2 days (48 hours). If an activity lasted for more than 5 minutes, it is considered to have lasted for 10 minutes, and if multiple activities were performed within 10 minutes, the activity that lasted for the longest period of time was recorded. These activities were classified into 9 large categories, 45 medium categories, and 153 small categories. Time was converted into minutes based on a single day (24-hour period), analyzed, and provided. We used data from family caregivers of dementia patients in this data.

10.7717/peerj.15178/supp-2 Supplemental Information 2 Codebook of raw data: 2019 time use survey

## Additional Information and Declarations

### Data Availability

The following information was supplied regarding data availability: The raw data is available in the Supplementary Files.
